# Practical lineshape of a laser operating near an exceptional point

**DOI:** 10.1038/s41598-021-85665-w

**Published:** 2021-03-17

**Authors:** Jinuk Kim, Juman Kim, Jisung Seo, Kyu-Won Park, Songky Moon, Kyungwon An

**Affiliations:** 1 Department of Physics and Astronomy and Institute of Applied Physics, Seoul National University, Seoul, 08826 Korea; 2 Faculty of Liberal Education, Seoul National University, Seoul, 08826 Korea

**Keywords:** Quantum optics, Optical sensors, Nonlinear phenomena

## Abstract

We present a practical laser linewidth broadening phenomenon in the viewpoint of high sensitivity of an exceptional point (EP). A stochastic simulation model is implemented to describe the fluctuations in the cavity resonance frequencies. The linewidth originated from external noises are maximized at the EP. The linewidth enhancement factor behaves similarly to the Petermann factor although the Petermann effect is not considered. In the long coherence time limit, the power spectral density of the laser exhibits a splitting in the vicinity of the EP although the cavity eigenfrequencies coalesce at the EP.

## Introduction

The exceptional point (EP) is a topological singular point in the parameter space at which two eigenstates coalesce due to non-Hermiticity^1^. It has recently attracted much attention in connection with many applications and fundamental issues such as directionality^2^, non-adiabaticity^3,4^, fractional topological charges^5^, anti-parity-time symmetric EP^6^, phonon lasing^7^, electromagnetically induced transparency at an EP^8^, and maximized entropy near an EP^9^. Another important issue is utilizing the EP to enhance the performance of a sensor^10,11^. Experimental confirmation of the high sensitivity at an EP has been done lately^12,13^. It has been adopted to enhance the Sagnac effect^14,15^ whereas there are conflicting theories about the existence of enhancement in the signal-to-noise ratio at the EP^16–19^. In addition, the relation between the fundamental sensitivity limit at an EP and the Petermann effect has been studied^20^. Recently, the idea of improving the sensitivity of the gyroscope with a mechanical EP has been proposed^21^ and sensitivity enhancement with exceptional surfaces has been demonstrated^22^.

The principle of enhancing the sensitivity is based on measuring the eigenvalue that is highly responsive to relatively slow external perturbations owing to its square-root-like variation near the EP with respect to system parameters. Narrow spectral width of the eigenvalue is thus required to increase the performance of a sensor. Moreover, gain or lasing can reduce the spectral width further and thereby facilitate resolving frequencies^10^. In order to utilize the high sensitivity of the EP in a microcavity laser, it is thus necessary to address the laser linewidth and line shape at an EP.

The practical linewidth of a laser is generally much larger than the theoretical limit given by the Schawlow–Townes formula because of background external disturbances^23^ such as mechanical as well as thermal fluctuations, pump power as well as phase fluctuations, etc. These background perturbations can introduce variation in the system parameters affecting the eigenvalues and thus the fluctuations in the laser frequency can also be amplified by the square-root-like structure near the EP, leading to linewidth broadening.

In addition, there is a fundamental linewidth broadening process called the Petermann effect arising from the non-orthogonality of the eigenstates of an open system^24,25^. Lasers are open systems and the laser linewidth broadening in this case is quantified by the Petermann excess noise factor. It is pointed out that the Petermann factor diverges at an EP^26^, where the eigenstate non-orthogonality is maximized. There are two issues in this regard. One is that under the fundamental linewidth broadening, the sensitivity or resolving power decreases due to the Peterman effect. The other is that the broadening due to the Peterman effect can be obscured by the practical linewidth broadening due to the background perturbations, unless the Peterman effect is greater than the latter.

In this paper, based on this perspective, we examine the linewidth broadening due to background external disturbances from the viewpoint of high sensitivity near an EP. The Petermann excess noise is not considered in our analyses. Parameter fluctuations are modeled with the Ornstein–Uhlenbeck process and the laser linewidth near an EP is obtained both numerically and analytically. It is found that the laser linewidth is broadened and maximized at the EP. Interestingly, although the Petermann excess noise is not included in our calculations, the broadening is approximately proportional to the Petermann factor under the condition of short correlation time of parameter fluctuations. Moreover, a splitting occurs in the lasing spectrum at the EP when the correlation time is long enough. Our results suggest that a linewidth broadening observed near an EP proportional to the Petermann factor does not necessarily mean the broadening must come from the Peterman effect. A sensor based on a microlaser can be designed and realized properly by considering the practical linewidth broadening effect considered in this paper.

## Results

### Eigenfrequency near an exceptional point

**Figure 1:**
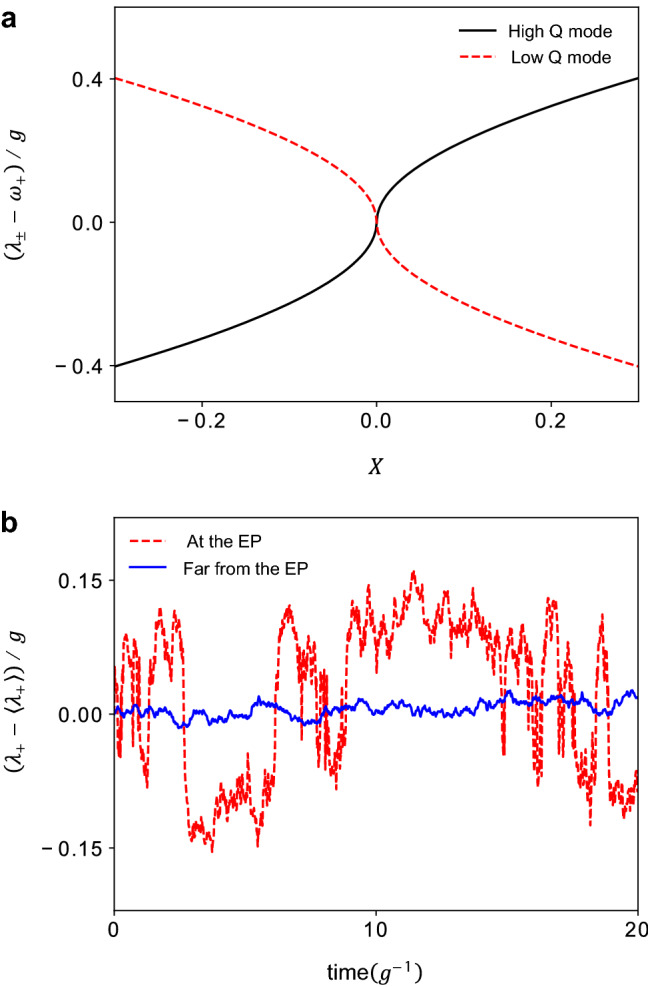
Eigenfrequencies and noise amplification near an EP. (**a**) Real parts of cavity resonance frequencies. Black solid and red dashed lines correspond to high- and low-Q modes, respectively. (**b**) Simulated frequency fluctuation of the high-Q mode at the EP (red dashed line, $\left\langle X\right\rangle =0.0$) and far from the EP (blue solid line, $\left\langle X\right\rangle =-2.0$). The standard deviation of the parameter *X* and the correlation time are assumed as follows: $\sigma _X=0.02,\tau _c = 1/g$.

Eigenfrequencies of interacting two lossy cavity modes can be described by the effective non-Hermitian Hamiltonian $(\hbar \equiv 1)$

1 $$\begin{aligned} \begin{pmatrix} \omega _1 -i \gamma _1&{} g \\ g &{} \omega _2 - i \gamma _2 \end{pmatrix}, \end{aligned}$$

where $\gamma _2 > \gamma _1$ and the diagonal terms represent complex resonance frequencies of non-interacting cavity modes whereas the off-diagonal terms are the coupling constant between them. In order to consider a system in the vicinity of an EP, a necessary condition for the EP is assumed: $(\gamma _2-\gamma _1)/ 2=g(>0)$. Then the eigenvalues are

2 $$\begin{aligned} \Lambda _\pm =\omega _+ - i (\gamma _1 + \gamma _2)/2 \pm \text {sgn}(X)g\sqrt{1+\left( \frac{X}{2}+i\right) ^2}, \end{aligned}$$

with $X\equiv (\omega _1-\omega _2)/g$ and $\omega _+\equiv (\omega _1 + \omega _2)/2$. Throughout this paper, the angular frequency and time are normalized with respect to *g* and $g^{-1}$, respectively. Real parts of eigenvalues are shown in Fig. 1a. The symbol $\lambda _+$ is the real part of the eigenvalue $\Lambda _+$of the high-Q mode which is dominant in the lasing signal to be considered below. The eigenvectors corresponding to $\Lambda _\pm$, respectively, are

3 $$\begin{aligned} \left| u_\pm \right\rangle = \begin{pmatrix} \frac{X}{2}+i\pm \text {sgn}(X)\sqrt{\left( \frac{X}{2}+i\right) ^2+1} \\ 1 \end{pmatrix}, \end{aligned}$$

which coalesce to a common eigenvector when *X* is equal to zero.

### Cavity parameter fluctuation model based on the Ornstein–Uhlenbeck process

External background disturbance such as mechanical as well as thermal fluctuations can be modeled by a stochastic Ornstein–Uhlenbeck process^27,28^. These external noises alter the refractive index or the geometry of the cavity and eventually the laser frequency. There have been attempts to explain the lineshape of conventional lasers with frequency fluctuations or external noises on the assumption that those follow the Ornstein–Uhlenbeck processes^29–31^.

**Figure 2:**
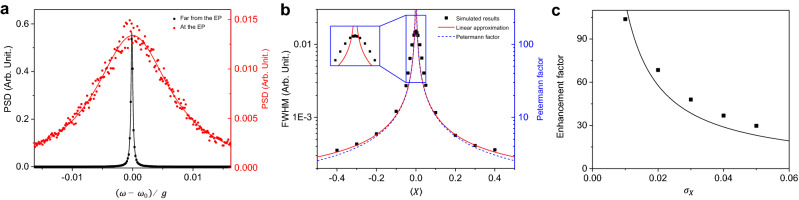
Laser lineshape, linewidth and its enhancement factor. (**a**) Red circles (black squares) represent the power spectral density (PSD) of the laser at the EP (far from the EP, $\left\langle X\right\rangle =0.4$). Red and black solid curves are Lorentzian fits. (**b**) Calculated linewidth (full width at half maximum or FWHM) by the analytic theory (red solid curve) under linear approximation and by the numerical simulation (black dots). The standard deviation of the parameter *X* and the correlation time are assumed as follows: $\sigma _X=0.02,\tau _c = 1/g, g\sqrt{\sigma _X}\tau _c\simeq 0.14$. The blue dashed curve represents the Petermann factor *K*. (**c**) The enhancement factor or the ratio of the linewidth at the EP to that far from the EP. The solid black line is a fit in the form of $y = \text {constant}/x$.

For simplicity, we focus on the high-Q cavity mode and assume that only the real part $\lambda _+$ of its resonance frequency fluctuates in time. The detuning parameter *X* is governed by the following stochastic differential equation:

4 $$\begin{aligned} dX=-\frac{1}{\tau _c}Xdt +\sqrt{D}dw, \end{aligned}$$

where $\tau _c$ is the correlation time of the fluctuation associated with the parameter *X*, *D* is the diffusion constant, and *w* denotes the Wiener process^32^. The parameter *X* obeys a Gaussian distribution, and its standard deviation $\sigma _X(=\sqrt{D\tau _c/2})$ is fixed throughout this paper except in Fig. 2c. With the parameter *X* stochastically varying, the fluctuation in the resonance frequency $\lambda _+$ is amplified at the EP as shown in Fig. 1b due to the square-root-like eigenvalue structure in the vicinity of the EP.

### Practical laser linewidth broadening

The resonance frequency fluctuatation is accumulated in the phase $\phi (t)$ of the laser field $E(t)=E_0e^{i[\omega _0t+\phi (t)]}$. From the frequency-noise spectral density, the autocorrelation function $G(\tau )$ of the laser, defined as $G(\tau ) \equiv \left\langle E^*(t)E(t+\tau )\right\rangle$, can be derived as follows^33,34^:

5 $$\begin{aligned} G(\tau )=\left| E_0\right| ^2e^{i\omega _0\tau }\exp \left[ -\frac{1}{\pi } \int _0^\infty {d\omega S_{\delta \omega }(\omega )\frac{\sin ^2\left( \frac{\omega \tau }{2}\right) }{\omega ^2}}\right] , \end{aligned}$$

where $E_0$ is the amplitude of the laser field, $\omega _0$ is the average value of laser frequency, and $S_{\delta \omega }$ represents the noise spectral density (see “Methods”). Except at EP, with a linear approximation, it can be simplified as

6 $$\begin{aligned} G(\tau )=\left| E_0\right| ^2 e^{i\omega _0\tau } \exp \left[ -C\left( \frac{|\tau |}{\tau _c}-1 + e^{-|\tau |/\tau _c}\right) \right] , \end{aligned}$$

where $C\equiv \frac{1}{2}\left( \left. \frac{d\lambda _+}{dX} \right| _{X=\left\langle X\right\rangle }\right) ^2\sigma _X^2 \tau _c^2$. The power spectral density (PSD) is then given by the Fourier transform of the autocorrelation function as

7 $$\begin{aligned} \begin{aligned} S(\omega )&= |E_0|^2\tau _c\left( \frac{e}{C}\right) ^C \{ C^{-i(\omega -\omega _0)\tau _c} \\&\quad \times \gamma \left( C+i(\omega -\omega _0)\tau _c,C\right) +c.c\}, \end{aligned} \end{aligned}$$

where $\gamma$ represents the lower incomplete gamma function, $\gamma (a,x)\equiv \int _0^x{e^{-t}t^{a-1}dt}$. Detailed calculations are given in “Methods”.

From the fluctuating laser frequency simulated as in Fig. 1b, the accumulated phase $\phi (t)$ of the laser field can be calculated numerically. By time-averaging the product of the electric field with a delayed copy of itself, a numerical autocorrelation function can also be obtained. Finally, the numerical lineshape is calculated by applying a fast Fourier transformation algorithm. This approach works even at EP. The results are shown in Fig. 2a. The lineshape becomes broader as we approach the EP. The linewidths obtained by numerical as well as analytic approximation methods are compared in Fig. 2b. They show similar behavior except in the vicinity of the EP. Exactly at the EP, the first-order approximation used in obtaining Eq. (6) fails and thus Eq. (7) is not valid. The accurate power spectral density at the EP will be discussed in a later section. Nevertheless, the numerical linewidth has a finite value at the EP in the example of Fig. 2a. The linewidth is roughly determined by the average slope in the fluctuation range of the parameter *X*. At the EP, the linewidth is about 100 times broader than those far from the EP in our calculation for the chosen parameters as shown in Fig. 2c. In order to observe a large enhancement factor, a small deviation of the parameter *X* is required. In the limit of small $\sigma _X$ (i.e., small noise amplitude), the enhancement factor is approximately proportional to $1/\sigma _X$ as shown in Fig. 2c.

### Relation to the Petermann factor in the vicinity of the EP

In this section, the Petermann factor and the practical linewidth broadening are compared. Adjoints of eigenmodes in Eq. (3) are given by

8 $$\begin{aligned} \left| \phi _\pm \right\rangle = \begin{pmatrix} \frac{X}{2}-i\pm \text {sgn}(X)\sqrt{\left( \frac{X}{2}-i\right) ^2+1} \\ 1 \end{pmatrix}. \end{aligned}$$

For small values of |*X*| $(\ll 1)$, the Petermann factor *K* is proportional to the inverse of the absolute value of the parameter *X*:

9 $$\begin{aligned} K=\frac{\left\langle \phi _+ | \phi _+ \right\rangle \left\langle u_+ | u_+ \right\rangle }{\left| \left\langle \phi _+ | u_+ \right\rangle \right| ^2} \simeq \frac{1}{\left| X\right| }. \end{aligned}$$

On the other hand, in the limit of small *C*, the incomplete gamma function in Eq. (7) can be simplified by using its series expansion^35^

10 $$\begin{aligned} \gamma (a,x)=x^a \sum _{n=0}^\infty {(-1)^n\frac{x^n}{n!(a+n)}}, \text {if } |x|<1, \end{aligned}$$

so the PSD in Eq. (7) approximately becomes a Lorentzian

11 $$\begin{aligned} S(\omega )\propto \frac{2C/\tau _c}{(\omega -\omega _0)^2+(C/\tau _c)^2}. \end{aligned}$$

Its FWHM linewidth $2C/\tau _c$ is proportional to the inverse of the absolute value of $\left\langle X\right\rangle$,

12 $$\begin{aligned} C\propto \left( \frac{d\lambda _+}{dX}\right) ^2\sim \frac{1}{\left| \left\langle X\right\rangle \right| }. \end{aligned}$$

Note that the linear approximation and the small *C* assumption fail exactly at EP as shown in Fig. 2b. Equation (11) is valid when $C\ll 1$ and $\left| \left\langle X\right\rangle \right| \gg \sigma _X$. Under this condition, the Petermann factor and the practical linewidth show similar dependence on $\left\langle X\right\rangle$ near the EP as shown in Fig. 2b.

Phase rigidity defined as the inverse square-root of the Petermann factor^36^, is a measure of the mixing of eigenstates. It vanishes at the EP where two eigenstates are maximally mixed to become one. Therefore, near an EP, the Petermann factor not only underlies the enhancement of fundamental laser linewidth but also implies how close the position in the parameter space is to the EP. On the other hand, due to the square-root-like structure of eigenvalues near the EP, the slope or the responsivity to external disturbances diverges. The slope is approximately proportional to the inverse phase rigidity near the EP. Consequently, the enhanced response broadens the linewidth in proportion to the slope squared. This makes the linewidth broadening due to external disturbances behave similarly to the Petermann factor.

**Figure 3:**
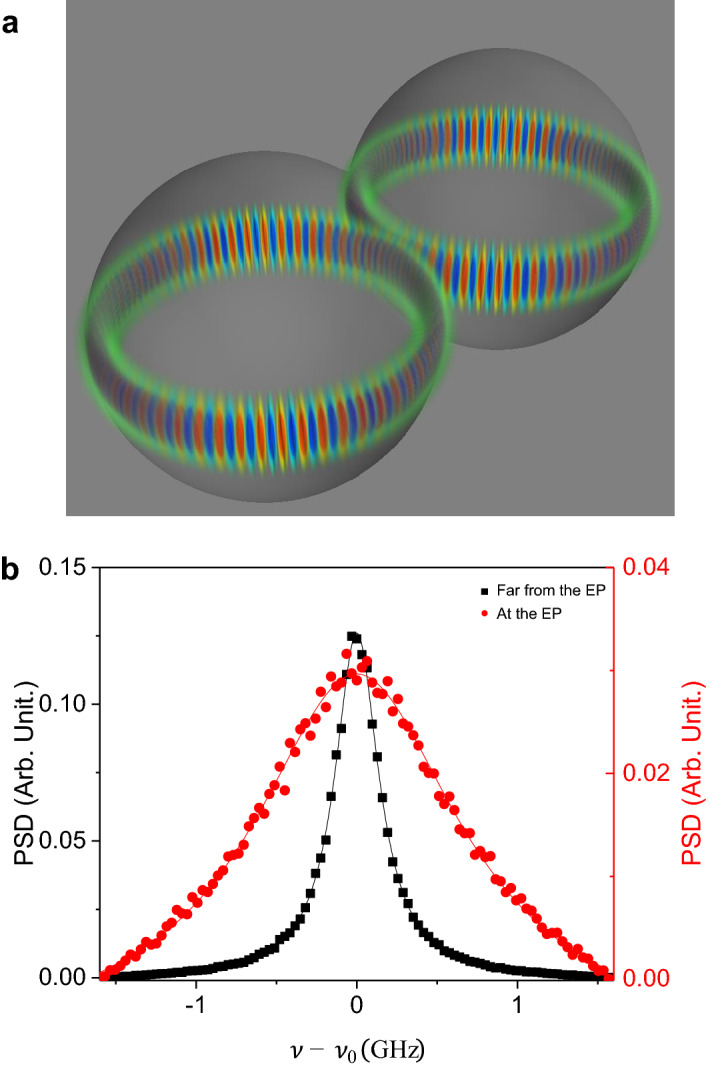
A laser made of coupled droplets as an example and its expected laser linewidth. (**a**) Schematic representation of coupled deformed droplets and two cavity modes forming an EP. (**b**) Red filled circles (black filled squares) represent PSD of the laser at the EP (far from the EP, $\left\langle X\right\rangle =-2.0$). The solid curves are Lorentzian fits. The standard deviation of the parameter *X* and the correlation time are assumed as follows: $\sigma _X=0.2,\tau _c = 1 \text {ps}=0.1/g, g\sqrt{\sigma _X}\tau _c\simeq 0.045$.

### Physical example: coupled deformed droplets

As a realistic example of the linewidth broadening effect discussed in the previous sections, two deformed dielectric droplets, which are radiatively coupled to each other, are considered. The material of the droplet cavity is assumed to be heavy water $(\text {D}_2\text {O})$ transparent to near infrared light^37^. The radii of droplets are about 10 $\upmu$m and the resonance wavelength is around 1450 nm. With this condition, the correlation time of thermally excited capillary waves on the surface is in the range of 0.1 to 1 ps^38^. Both droplets are slightly deformed in order to lift the degeneracy in azimuthal modes as well as to induce the imaginary parts of individual modes to be different. For these purposes, any other techniques such as loss control by a nano tip can also be applied^39^. Droplets and the transverse electric eigenmode are depicted schematically in Fig. 3a.

These droplets are known to vibrate constantly due to thermally excited capillary waves on the surface^38,40^. Spectral linewidth $\Delta \nu$ (half width) originated from such vibrations has been studied, and it is in the order of

13 $$\begin{aligned} \Delta \nu \sim \frac{\omega _0}{8\pi a}\sqrt{\frac{k_BT}{4\pi \sigma }\ln {\left( \frac{2a^2}{3d^2}\right) }}, \end{aligned}$$

where *a* is the radius, $k_B T$ is the product of the Boltzmann constant and the temperature, $\sigma$ represents the surface tension, and $\pi d^2$ is the effective area per molecule^40^. At room temperature under the condition mentioned above, the half width at half maximum given by Eq. (13) far from the EP, is about 3 GHz. Using the coupling constant $g/2\pi$ of 16 GHz, reported for cavities of similar sizes^41^, we obtain $\sigma _X\simeq$ 3 GHz/16 GHz $\sim$ 0.2 of our model. With the known correlation time $\tau _c\sim$ 1 ps, we obtain about fivefold spectral broadening at the EP as shown in Fig. 3b. Note this linewidth enhancement is of purely classical origin.

**Figure 4:**
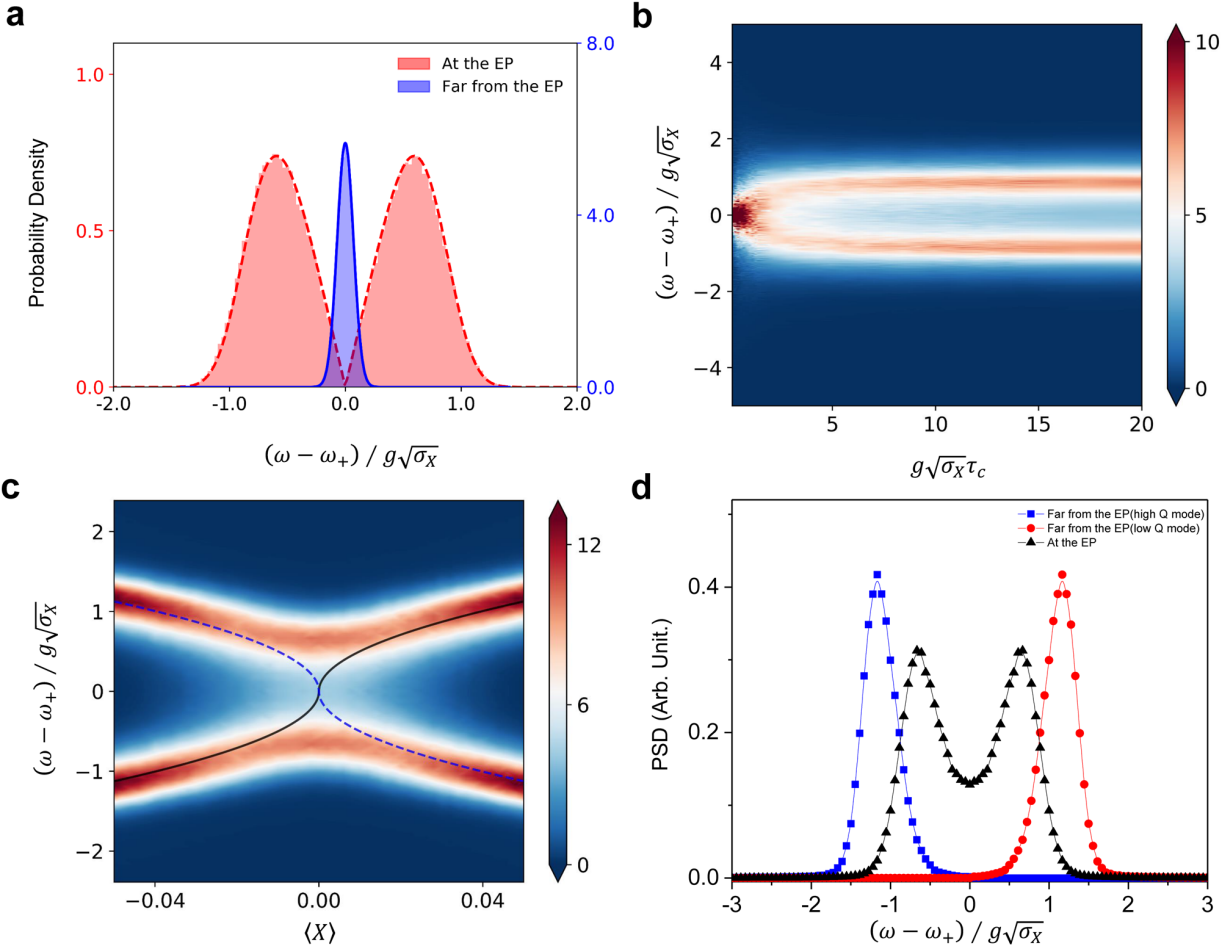
Splitting in the distribution of the resonance frequency and the resulting split laser spectrum at the EP. (**a**) Probability density of the resonance frequency of the single cavity mode at the EP (red) and far from the EP (blue). The blue solid (red dashed) curve represents the probability density of (the square root of) the Gaussian variable. (**b**) PSD of the laser at the EP as the correlation time $(\tau _c)$ is varied. (**c**) Two dimensional contour map of PSD. The black solid and blue dotted curves are the real parts of the cavity eigenfrequencies in Fig. 1a, corresponding to high Q and low Q, respectively. The standard deviation of the parameter *X* and the correlation time are assumed as follows: $\sigma _X=0.02, \tau _c = 200/g, g\sqrt{\sigma _X}\tau _c\simeq 28.3$. (**d**) PSD of the laser at the EP (black triangles) and far from the EP (blue rectangles and red circles, $\left\langle X\right\rangle =-0.05$). For the latter, the low Q (on the right) and high Q (on the left) are well separated while at the EP such distinction is impossible.

### Splitting of the spectrum at the EP in the limit of long correlation time

Gaussian external noise is assumed throughout this paper. Far from the EP, the resonance frequency of the single cavity mode also obeys a normal distribution due to approximately linear response. However at the EP$(\left| \left\langle X\right\rangle \right| \ll \sigma _X)$, the linear approximation fails, as discussed before, and thus Eq. (11) is not valid anymore. Instead, because of the diverging slope, a dip occurs at the center of the resonance frequency distribution induced by the fluctuating *X*. This can be seen by considering that the resonance frequency near the EP is approximated as $\lambda _+\simeq \omega _+ + \text {sgn}(X)g\sqrt{|X|/2}$. Because of the sign dependence, the resonance frequency distribution due to the fluctuating *X* splits into two groups although $\lambda _\pm$ coalesce at the EP. The probability density or the resonance frequency distribution numerically obtained is depicted in Fig. 4a with the approximate analytic probability density function $\sqrt{\frac{8}{\pi }}\frac{| \omega - \omega _+ |}{g\sqrt{\sigma _X}}e^{-2(\omega - \omega _+)^4/(g^4\sigma _X^2)}$ (see “Methods” for detailed calculations).

The frequency separation between the two peaks is about $g\sqrt{\sigma _X}$. If the correlation time $\tau _c$ of the external noise is larger than $\left( g\sqrt{\sigma _X}\right) ^{-1}$, which is the beating period of the two frequencies, there can be a splitting in the PSD at the EP. In the opposite limit, i.e., when $g\sqrt{\sigma _X}\tau _c\ll 1$, the PSD is single peaked. This is the case for the parameter sets in Figs. 2 and 3. As depicted in Fig. 4b, a single-peak laser spectrum splits into two peaks as the correlation time is increased. The distance between two peaks is almost the same as that of the probability density of the cavity frequency.

So far, we have neglected the low Q mode of eigenfrequency $\lambda _-$. In the vicinity of the EP, however, the Q factors of two modes become similar and thus the low-Q mode should be also considered. For simplicity in analysis, we assume that the powers (i.e., the integration of PSD over frequency) of two modes are the same, which is valid far above the laser threshold. Both eigenvalues exhibit the square-root-like structure, and hence the splitting of the PSD occurs for both. This leads to a splitting in the laser spectrum across the EP as we scan $\langle X\rangle$ although the eigenfrequencies $\lambda _\pm$ coalesce there ($\langle X\rangle =0$) as shown in Fig. 4c. The cross sections of the contour plot at the EP and far from the EP are depicted in Fig. 4d.

## Discussion

In the short correlation time limit, as in Fig. 2b, the Petermann factor and the practical linewidth show similar dependence on $\left\langle X\right\rangle$ near the EP. Such similarity is totally unexpected because the origins of two mechanisms are fundamentally different. The Petermann effect amplifies intrinsic quantum noise, leading to the broadening of the Schawlow–Townes linewidth^25^, whereas the broadening mechanism in this paper comes from the amplification of the background external noise. The fact that both linewidths are proportional to the Petermann factor suggests that observation of laser linewidth broadening proportional to the Petermann factor does not necessarily mean the broadening is due to the Petermann effect.

One way of distinguishing the mechanisms of the observed linewidth broadening, whether the Petermann effect or classical fluctuations, is to investigate the power dependence of the broadening. If it comes from the Petermann effect, the linewidth broadening would be inversely proportional to the laser output power, just like the Schawlow–Townes linewidth. If the broadening is induced by the external noise with the linewidth much larger than the Schawlow–Townes linewidth, there would be no such power dependence. Another way of distinguishing the broadening mechanisms is to investigate photon statistics below lasing threshold. For the Petermann effect, the correlation time in the second order correlation $g^{(2)}(t)$ of the output photons would be in the order of the dephasing time of the lasing transition of the gain medium. For the background external noise, the correlation time in $g^{(2)}(t)$ would be that of the external noise. Since enhancement factors of both mechanisms have the same order of magnitude, the predominant linewidth would be determined by the details of the system.

Since we assume lasing far above threshold, the integrated powers for low and high Q modes in Fig. 4b,c are the same. Their linewidths are determined by the slopes of the resonance frequencies as seen in Figs. 1a and 4c. For a more realistic analysis, the fluctuation of the two mode frequencies should be calculated independently and a multimode laser theory^25,42^ should be considered under variable pumping strength.

The condition for the correlation time for observing the splitting is experimentally feasible. For example, a numerical calculation predicts that the correlation time of a 1mm water droplet is about 10 ps^38^, making $g\sqrt{\sigma _X}\tau _c\sim 0.45$. Furthermore, the correlation time for capillary waves on the colloidal liquid-gas surface longer than a few seconds has been reported^43^. By using stable solid such as ultralow expansion glass^44^ instead of liquid, the thermal fluctuation discussed above can be suppressed. In this case, a few-second-long correlation time of mechanical noise^45^ is possible to allow observation of the splitting at the EP.

To summarize, we investigated the practical lineshape of a laser operating in the vicinity of an EP formed by two interacting cavity modes. A stochastic simulation model was implemented to describe the fluctuations in the cavity resonance frequencies. The linewidth of the laser was broadened due to the increased sensitivity near the EP and exhibited a finite peak value at the EP. The linewidth showed a parameter dependence similar to the Petermann factor although the Petermann excess noise was not considered in our analysis. In this regard, a linewidth broadening proportional to the Petermann factor does not necessarily come from the Petermann effect. With a long correlation time of external noises, there was a splitting in the power spectral density although the cavity eigenfrequencies coalesce at the EP. Our result can be used to evaluate practical performance of sensors based on the EP phenomenon.

## Methods

### Noise spectral density of the frequency fluctuation

To obtain an approximate analytic expression of the noise spectrum, Eq. (2) is expanded to first order at the point $X=\left\langle X\right\rangle$:

14 $$\begin{aligned} \Delta \lambda _+ \simeq \left. \frac{d\lambda _+}{dX} \right| _{X=\left\langle X\right\rangle }\Delta X, \end{aligned}$$

where $\left\langle X\right\rangle$ represents the average value of *X* and $\Delta \lambda _+$ and $\Delta X$ are deviations from their average values, respectively. According to the Ornstein–Uhlenbeck theory, the correlation function of the frequency noise can be expressed as

15 $$\begin{aligned} G_{\delta \omega }(\tau )=\left\langle \Delta \lambda _+(t)\Delta \lambda _+(t+\tau )\right\rangle =\frac{2C}{\tau _c^2}e^{-\left| \tau \right| /\tau _c}, \end{aligned}$$

where $$C\equiv \frac{1}{2}\left( \left. \frac{d\lambda _+}{dX} \right| _{X=\left\langle X\right\rangle }\right) ^2\sigma _X^2 \tau _c^2$$.

Exactly at EP, *C* diverges and thus Eq. (15) is not valid there. Except at EP, by the Wiener-Khinchin theorem, the noise spectral density can be calculated by taking the Fourier transform of the correlation function.

16 $$\begin{aligned} S_{\delta \omega }(\omega )=\int _{-\infty }^\infty {G_{\delta \omega }(\tau )e^{-i\omega \tau }d\tau }=\frac{4C}{\tau _c}\frac{1}{1+(\omega \tau _c)^2}. \end{aligned}$$

### Correlation function and the power spectral density calculation

After substituting Eq. (16), which is valid except at EP, into the exponent in Eq. (5), the integral can be divided into three terms as follows:

17 $$\begin{aligned} \begin{aligned} \int _0^\infty {S_{\delta \omega }(\omega )\frac{\sin ^2(\omega \tau /2)}{\omega ^2}d\omega }&= 4C \int _0^\infty {\frac{\sin ^2{(\omega \tau /2})}{(\omega \tau _c)^2}d(\omega \tau _c)} \\&\quad - 2C\int _0^\infty {\frac{1}{1+(\omega \tau _c)^2}d(\omega \tau _c)} \\&\quad + 2C\int _0^\infty {\frac{\cos (\omega \tau )}{1+(\omega \tau _c)^2}d(\omega \tau _c)}. \end{aligned} \end{aligned}$$

By using the following definite integral formulae^46^,

18 $$\begin{aligned} {} & \int _0^\infty {\frac{1}{1+x^2}dx} = \frac{\pi }{2}\\&\int _0^\infty {\frac{\sin ^2{px}}{x^2}dx} = \frac{\pi |p|}{2}\\&\int _0^\infty {\frac{\cos {mx}}{x^2+a^2}dx} = \frac{\pi }{2|a|}e^{-|ma|}, \end{aligned}$$

Eq. (17) becomes

19 $$\begin{aligned} \int _0^\infty {S_{\delta \omega }\frac{\sin ^2(\omega \tau /2)}{\omega ^2}d\omega } =\pi C\left( |\tau |/\tau _c-1+e^{-|\tau |/\tau _c}\right) . \end{aligned}$$

One can obtain the correlation function, Eq. (6), by substituting this into Eq. (5). The power spectral density is the Fourier transform of the result,

20 $$\begin{aligned} \begin{aligned} S(\omega )&=\int _{-\infty }^\infty {G(\tau )e^{-i\omega \tau }d\tau } \\&= |E_0|^2 \int _{-\infty }^\infty {e^{-i(\omega -\omega _0)\tau }\exp \left[ -C\left( |\tau |/\tau _c-1+e^{-|\tau |/\tau _c}\right) \right] d\tau } \\&= |E_0|^2 e^C \left\{ \int _{0}^\infty {e^{-C\tau /\tau _c-i(\omega -\omega _0)\tau }\exp \left[ -Ce^{-\tau /\tau _c}\right] d\tau }+c.c\right\} . \end{aligned} \end{aligned}$$

With a substitution $x\equiv C e^{-t/\tau _c}$, the integral can be evaluated as

21 $$\begin{aligned} S(\omega ) =|E_0|^2 \tau _c \left( \frac{e}{C}\right) ^C\left( C^{-i(\omega -\omega _0)\tau _c} \int _{0}^C{x^{C+i(\omega -\omega _0)\tau _c-1}e^{-x}dx}+c.c\right) . \end{aligned}$$

By the definition of the incomplete gamma function, this leads to Eq. (7).

### Approximate probability density function of the resonance frequency at the EP

A variable *Y* is defined and approximated at the EP as

22 $$\begin{aligned} Y\equiv (\lambda _+ - \omega _+ )/g\sqrt{\sigma _X}\simeq \text {sgn}(X)\sqrt{|X|/2\sigma _X}. \end{aligned}$$

It is the square root of a Gaussian random variable $(\sim {\mathcal {N}}(0,1/4))$, and obeys a square-normal distribution. The cumulative distribution function of *Y* can be obtained by integrating the normal probability density function,

23 $$\begin{aligned} P(Y \le y)=\int ^{\text {sgn}(y)y^2}_{-\infty }{d\xi \sqrt{\frac{2}{\pi }}e^{-2\xi ^2} }, \end{aligned}$$

where $y\equiv (\omega - \omega _+)/g\sqrt{\sigma _X}$. By differentiating Eq. (23) to *y*, the probability density function of *Y* is determined as

24 $$\begin{aligned} P(y)= \sqrt{\frac{8}{\pi }}|y|e^{-2y^4}, \end{aligned}$$

which is drawn in Fig. 4a.

## Data Availability

The datasets generated during the current study are available from the corresponding author on reasonable request.

## Code availability

The code that supports the findings of this study are available from the corresponding author upon reasonable request.
